# Volcanic Island lightning prebiotic chemistry and the origin of life in the early Hadean eon

**DOI:** 10.1038/s41467-023-37894-y

**Published:** 2023-04-10

**Authors:** Jeffrey L. Bada

**Affiliations:** Scripps Institution of Oceanography, University of California, San Diego, La Jolla, CA 92024 USA

**Keywords:** Geochemistry, Astrobiology, Origin of life

## Abstract

The early Hadean eon (>4Ga) may have had a periodically ice-covered global ocean and limited subaerial landmass, and this could have resulted in infrequent lightning occurrence. This infrequency of lightning may have limited the synthesis of prebiotic compounds necessary for life’s origins. Here I present a hypothesis that lightning associated with volcanic island eruptions created focal points for the generation of prebiotic ingredients and ultimately the origin of life.

## Volcanic lightning

Lightning is often generated within ash clouds during volcanic eruptions, having been reported at nearly 400 eruptions of 152 volcanoes^1^. Volcanic lightning puts on spectacular displays that have been noted throughout history. Gaius Plinius Caecilius Secundus (Pliny the Younger) in 79 CE described “long fiery shapes, similar to lightning, only bigger” when describing the Mt. Vesuvius eruption that obliterated Pompeii^2^. On 26 August 1883, several sailing ships were near the volcano on Krakatoa (Indonesia) when it erupted in one of the most violent eruptions in the modern era^3^. The commander Sir Robert Sale noted in the ship’s log “a dreadful black cloud was torn by gushing flames and great tongues of fire like much-magnified lightning”. In 1985, the American pop art icon Andy Warhol spent time in Naples, where he created 18 paintings of his rendition of Vesuvius erupting with what appear to be depictions of intense lightning^4^_._

A recent example^5^ of an explosive volcanic island eruption and its associated intense lightning is the massive Plinian eruption of the Hunga Tonga-Hunga Ha’apai (Indonesia) subsurface volcano (~150 m below sea level) that took place on 15 January 2022. This explosive eruption rated at least 5 on the Volcanic Explosive Index and was heard up to 10,000 km away. It sent ash, gases, and vaporized sea water in a plume reaching over 50 km into the mesosphere. These components represented the perfect combination of ingredients needed for the electrification of the plume and, thus, the generation of lightning^6^. The result was a stunning 25,508 lightning strikes in 5 min (85/sec), which equaled 170% of the total number of strikes on the Earth at that time^5^.

## Rarity of lightning over the ocean

On Earth today^7^, the lightning land/ocean ratio is 10/1. The lower level of lightning over the oceans is caused by injection in the form of sea spray of coarse marine aerosols (>1 µm) into clouds, in contrast to the finer aerosols (<1 µm) injected on land^8^. The finer aerosols over land promote greater electrification of the clouds; the net result being more lightning over land.

Lightning is thought to have played an important role in the synthesis of organic compounds required for the origin of life^9^. While scant actual evidence survives for Hadean surface conditions, there is some suggestion of a ‘water world’ scenario, where the amount of exposed sub-aerial land was perhaps only ~12% compared to today’s 29%^10^. Moreover, these speculative early oceans may have been periodically ice covered^11^ creating intermittent ‘Snowball’ Earth conditions. Lightning in the Earth’s polar regions today is virtually non-existent^12^. These combined factors have important potential implications concerning lightning on the Hadean Earth. With the scarcity of exposed subaerial continental areas, and an episodically ice-covered early ocean, would the amount of lightning have been rare?

This presents a conundrum: Under this scenario, would the production of prebiotic organic compounds considered crucial for the origins of life have been limited?

## Hypothesis: Hadean eon volcanic island lightning was crucial in the production of prebiotic organic compounds and in turn the origin of life

**Fig. 1:**
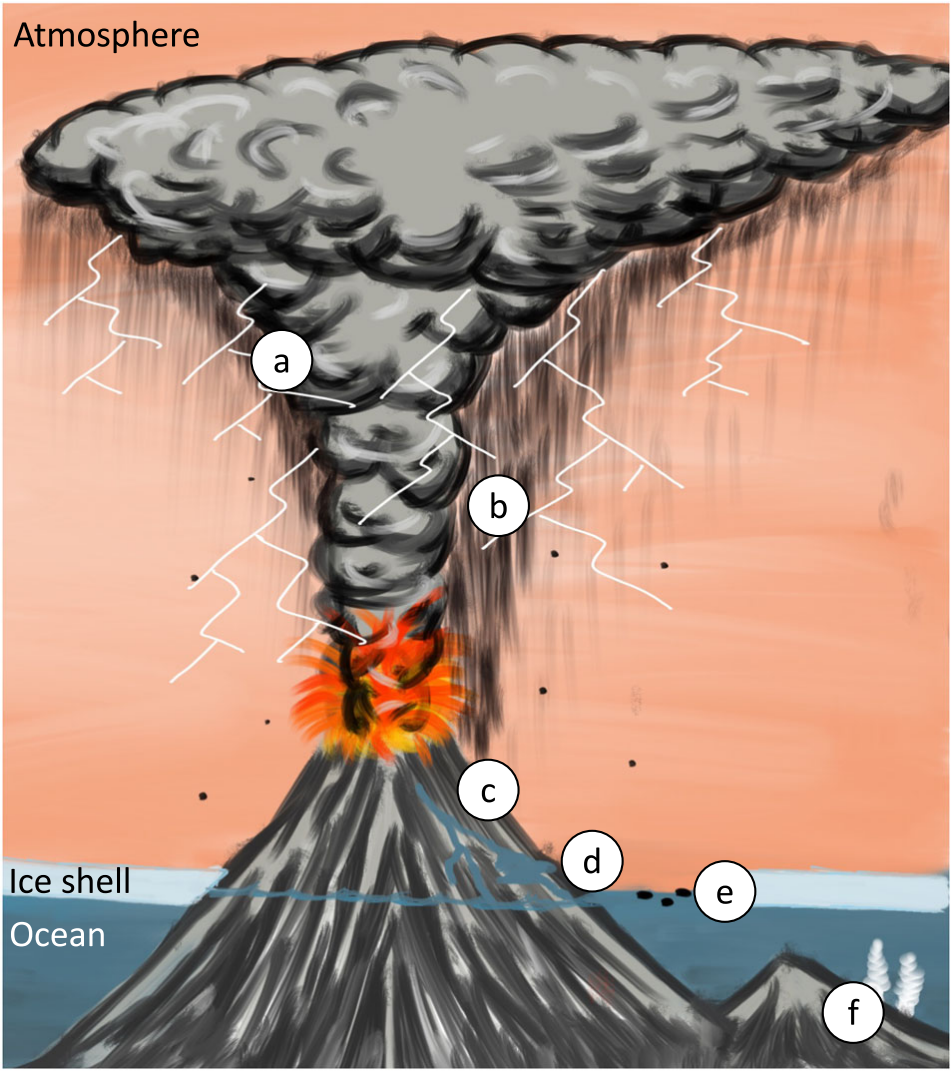
Schematic of the hypothesized Hadean volcanic island setting, erupting through the ice, generating lightning and subsequent prebiotic chemistry. **a** Volcanic lightning generates prebiotic reagents; **b** Condensation and rain out of organic products; **c** Run off, concentration in ponds, warm/dry cycles encourage reactions; **d** Tidal action where ice shell is discontinuous, washing into the sub-ice ocean; **e** pumice rafts transporting adsorbed reagents; **f** Further processing in hydrothermal setting forming more complex organic compounds. Image credit Samuel Royle.

With a periodically ice-covered Earth and limited subaerial land areas, lightning frequency may have been significantly reduced in comparison to today. However, increased plume-related magmatic activity on the young Earth^13^ implies that hot spot volcanism would have been present on the early Hadean Earth, with volcanic islands poking out above the putative ocean/ice surface. Therefore, hot spot volcanic islands may have provided an important lightning source, and in turn been a focal point for the generation of the ingredients and building blocks necessary for the origins of life^14–19^ (Fig. 1).

The erupting island volcanoes accompanied by intense lightning would have expelled water and gases into an episodically reducing atmosphere^18^, providing ideal conditions for prebiotic syntheses. With the reduced gases and lightning, important prebiotic reagents such as hydrogen cyanide, phosphide, aldehydes and ketones as well as some complex compounds could have been generated^9^.

Some laboratory studies have been conducted to investigate the potential of prebiotic synthesis by volcanic lightning. A version of the Miller apparatus in which a plume of water vapor was injected into a spark in the presence of reduced gases yielded a rich variety of amino acids^19^. Another experiment was conducted using a volcanic lightning simulator with rinsed volcanic ash collected from the 2013 Sakurajima volcano eruption in Japan, along with various reducing gas mixtures^16,17^. Preliminary results indicated that some amino acids (glycine, nearly-racemic D- and L-alanine, β-alanine) were produced in this experiment. These combined experiments support the hypothesis that volcanic lightning played an integral role in early Hadean prebiotic synthesis.

The discharged water from eruptions would have produced lakes, pools, and ponds on the volcano flanks^16,20^. In addition, there would have been volcanic rock pools along the volcano/ocean shoreline where seawater and rainwater could have collected and been periodically flushed. In these various watery environments, temperatures could cycle between cool and warm conditions depending on geothermal heat input and meteorological conditions. These temperature cycling events, along with evaporative wet/dry cycles^21,22^, have been shown to promote important prebiotic polymerization reactions of amino and nucleic acids.

Explosive eruptions can produce copious amounts of pumice. For example, the eruption of an unnamed volcano in the Hunga Tonga-Hung-a Ha’apai archipelago on 7 August 2019 produced a huge amount of pumice^23^, which quickly coagulated into a 195 km^2^ floating pumice raft. Evidence that pumice was present on the early Earth is provided by its occurrence in the 3460 Ma Apex Basalt in Western Australia^24^. With its highly porous structure, it could be speculated that Hadean pumice could have absorbed prebiotic reagents and other products^25^ associated with volcanic island eruptions, dispersing these throughout the oceans^26^ and seeding newly emerged continents.

In the Hadean, pumice-based transport processes could have seeded newly emerging continents. There, periodic temperature and evaporative wet/dry cycles could take place allowing for further prebiotic processing and molecular evolution to occur. As the products of these reactions became increasingly complex, eventually, by chance, a “self-sustaining chemical system capable of Darwinian evolution was produced^27^. This would have marked both the start of evolution and the appearance of life.

According to this hypothesis, eruptions of volcanic islands on the primitive Earth with their associated intense lightning along with pumice may have played a major role in the prebiotic chemistry of the early Hadean and even possibly the origin of life. Whether this actually occurred is unknown due to the paucity of the rock record going this far back in time, with the Hadean “Waterworld” or “Snowball” hypotheses being perhaps the most uncertain^28^.

If, where and when the emergence of life took place in this scenario requires further refinement of the underlying mechanisms and processes. But one thing is certain: *Thunder is good, thunder is impressive; but it is lightning that does the work.*- Mark Twain.
